# The impact of physical exercise on adolescent psychological resilience: an empirical analysis based on deep neural networks

**DOI:** 10.3389/fpsyg.2025.1709478

**Published:** 2026-01-06

**Authors:** Yuehao Chen, Jiulong Song, Guoguo Zhao, Jian Fu

**Affiliations:** School of Physical Education, Yangzhou University, Yangzhou, Jiangsu, China

**Keywords:** adolescents, deep neural network, nonlinear relationship, physical exercise, psychological resilience, SHAP model

## Abstract

**Objective:**

The junior high school years represent a crucial period in adolescents’ physical and psychological development, during which mental health concerns have gained significant attention. Psychological resilience, a modifiable protective factor, is vital for maintaining mental wellbeing.

**Method:**

This study involved 1,064 junior high school students, who were selected through stratified random sampling. Data were collected using the Physical Activity Rating Scale-3 (PARS-3) and the Adolescent Resilience Scale. The analysis integrated multiple linear regression, deep neural networks (DNN), and the SHAP (Shapley Additive Explanations) model for interpretability.

**Results:**

Physical exercise intensity, duration, and frequency were significantly positively correlated with adolescents’ psychological resilience and its subdimensions. Notably, the interaction between intensity and duration was prominent, exhibiting nonlinear effects. Furthermore, the interaction between grade level and physical exercise was statistically significant, highlighting lower grades as a key intervention period. For individuals with low psychological resilience, increasing both exercise intensity and duration led to more effective intervention outcomes.

**Conclusion:**

Physical exercise is significantly positively associated with adolescent psychological resilience, and this association may operates through potential nonlinear pathways. Furthermore, exercise characteristics may interact with demographic variables such as grade level, providing empirical insights to inform the development of tailored intervention strategies.

## 1 Introduction

### 1.1 The role of psychological resilience in adolescent mental health

Adolescence is a key developmental stage characterized by rapid physiological growth (Zheng and Wang, 2013) and delayed psychological maturation (Yao and Sun, 2008), increasing vulnerability to anxiety and depression. Epidemiological studies indicate that the prevalence of psychological issues among adolescents ranges from 24.6 to 45.2% (Jiang, 2016), making adolescent mental health a global public health priority (Kieling et al., 2011; Zheng, 1996). Extensive research has confirmed that psychological resilience—an adjustable trait—exhibits a significant positive correlation with mental health (Liu et al., 2019; Zeng et al., 2014; Song G. W. et al., 2014, 2013; American Psychology Association Help Center, 2004; Li and Zhang, 2011). As an intrinsic protective mechanism (Hu and Gan, 2008), resilience enables individuals to cope with adversity and mitigate psychological distress (Zhou et al., 2013). Notably, psychological resilience is not static but a dynamic trait shaped by both positive (Cioffi et al., 2010) and negative environmental factors (Liang et al., 2019)—a process particularly influenced by adolescent physical exercise.

### 1.2 The impact of physical exercise on psychological resilience and its interaction effects

Physical exercise, as a cost-effective and easily implementable protective factor (Yang et al., 2021), can enhance psychological resilience through mechanisms such as neural remodeling, emotion regulation (Zhang et al., 2022), and optimization of brain circuit functions. Neuroimaging studies show that physical activity enhances self-regulation-related neural circuits, such as the prefrontal cortex, while aerobic exercise improves cognitive control networks (Krafft et al., 2014), offering neural mechanistic support for the link between moderate-to-high intensity, regular-frequency exercise, and resilience (Belcher et al., 2021). Specifically: high-intensity exercise activates the sympathetic nervous system, stimulating the release of endorphins and serotonin to regulate emotional processing pathways (Abdulaziz Muhsen and Abdulaziz Muhsen, 2020), aligning with the need for anxiety relief in emotional control (Dunston et al., 2022); high-frequency exercise boosts self-efficacy via “goal-feedback” cycles (Donnelly et al., 2016); and moderate-duration exercise strengthens self-regulation (Baumeister and Vohs, 2016)—with effects enhanced when matched with appropriate intensity (Eston et al., 2012). Additionally, interactions between exercise characteristics (e.g., intensity × duration) may yield synergistic effects (Parfitt and Hughes, 2009), further indicating that exercise’s impact on resilience is non-linear. This protective effect is also cross-culturally consistent: a study on young e-sports players in the Middle East (Solmaz et al., 2025) confirms its universal value for adolescent well-being, underscoring exercise’s consistent role in boosting adolescent resilience across cultural contexts.

Recent studies (Wu et al., 2023) also suggest that the impact of physical exercise on psychological resilience is moderated by demographic factors, including gender, grade level, and only-child status. For example, males tend to engage in longer exercise durations, with stronger links between physical activity and stress-resilience outcomes, and a significant gender × exercise frequency interaction effect (Peyer et al., 2022; Brittain et al., 2022). At the grade level, adolescence is a crucial period for resilience development, with resilience levels following distinct developmental trajectories across age groups (Xing et al., 2023). Additionally, only children may face unique challenges due to differences in family resource allocation (Killgore et al., 2020).

### 1.3 The value of the DNN-SHAP model in nonlinear relationships

Most existing studies implicitly assume a linear relationship between physical exercise and psychological resilience, failing to capture the complex nonlinear dynamics between exercise intensity, duration, frequency, and resilience. These studies generally over-rely on linear frameworks, which not only overlook the intricate nonlinear interactions among exercise characteristics but also downplay the potential role of demographic variables.

To address these limitations, deep neural networks (DNN)—with their multi-layered structure—can capture nonlinear patterns between variables, offering a novel approach for analyzing the complex relationships between physical exercise and psychological constructs (Dong et al., 2023). However, DNN’s lack of interpretability severely hinders the translation of results: while it may confirm that exercise characteristics are related to resilience, it cannot clarify how specific exercise features (or their interaction with grade) influence resilience changes. This “black box” problem makes it difficult to translate research findings into targeted exercise interventions for adolescents of different grades—an urgent need for addressing adolescent mental health challenges.

Thus, integrating DNN with interpretable tools is essential. The SHAP model, based on game-theoretic Shapley values, quantifies the contribution of individual features to predictive outcomes while upholding the principles of “local accuracy,” “missingness,” and “consistency” (Lundberg et al., 2020). Increasingly applied in sports science and psychology to resolve machine learning interpretability issues (Yan and Lu, 2025), the DNN-SHAP integration will not only accurately capture nonlinear relationships but also overcome the “black box” dilemma—providing empirical support for developing scientific exercise intervention strategies to enhance adolescent psychological resilience.

### 1.4 Research hypotheses

Existing research provides important theoretical and empirical support for formulating the hypotheses of this study. Theoretically, the theory of adolescent psychological development (Mastorci et al., 2024) points out that adolescent psychological development does not follow a linear evolutionary path but adheres to a nonlinear trajectory of “weak promotion—fluctuation—weak inhibition,” which offers a core theoretical basis for understanding the developmental patterns of adolescent psychological resilience. Empirically, the impact of exercise characteristics on resilience exhibits a significant “threshold effect”: for instance, low-frequency exercise has no significant impact on psychological resilience, moderate-frequency exercise yields the strongest promotional effect, while excessively high-frequency exercise may instead inhibit psychological resilience due to issues such as fatigue (Dunston et al., 2022).

Based on the above theoretical foundations and empirical findings, and in response to the core research questions of this study, the following research hypotheses are proposed: H1: The intensity, duration, and frequency of physical exercise are significantly positively correlated with adolescent psychological resilience and its five subdimensions. Furthermore, the strength of these relationships varies under different levels of exercise intensity, duration, and frequency. H2: Gender, grade level, and only-child status will interact with exercise characteristics to collectively influence adolescent psychological resilience and its subdimensions. H3: The SHAP model can quantify the contributions of exercise characteristics and demographic variables to psychological resilience and its subdimensions, and reveal how the interactions between these variables influence resilience outcomes.

## 2 Research objects and methods

### 2.1 Research objects

Sample size estimation was conducted using a validated formula from prior literature (Cochran, 1977): The required sample size *n* was calculated using the formula in Equation 1,


(1) $$n=\frac{z^{2}\cdot p\cdot (1-p)}{e^{2}}$$

where *n* is the required sample size, *z* is the critical value corresponding to the desired confidence level, *p* is the estimated proportion, and *e* is the target sampling error. For this study, *z* was set at 1.96 (corresponding to a 95% confidence interval), *p* was conservatively estimated at 0.50, and *e* was set at 0.03.

This resulted in a theoretical minimum effective sample size of 1,067. Using stratified random sampling, participants were recruited from seven middle schools across Jiangsu Province (Nantong, Wuxi, Yancheng, Xuzhou, Taizhou, Nanjing), as well as one middle school each in Changsha and Zhengzhou. Questionnaires were distributed through both online (Wenjuanxing platform) and offline (paper-based) methods.

Before data collection, participants were fully informed about the study’s purpose to ensure informed consent, and investigators underwent standardized training to maintain data quality. With assistance from homeroom and subject teachers, participants completed the questionnaires anonymously. On-site distribution and collection were carried out by trained investigators. Initially, a total of 408 paper questionnaires and 845 online questionnaires were collected. Invalid paper questionnaires (characterized by incomplete or erroneous responses) were excluded. Online questionnaires were further screened based on completion time, with responses completed in less than 10 min deemed invalid. After excluding 189 invalid questionnaires, 1,064 valid responses were retained, resulting in an effective recovery rate of 84.9%.

### 2.2 Outcome measures

#### 2.2.1 Physical Activity Rating Scale-3 (PARS-3)

The Physical Activity Rating Scale-3 (PARS-3) (Liang, 1994), revised by Liang Deqing and other scholars based on the international sports assessment system for localization adaptation, consists of 3 core items and evaluates three core dimensions of exercise behavior: intensity, duration, and frequency. Specifically, intensity is measured on a 5-point scale, frequency is quantified as weekly participation times on a 5-point scale, and duration records single-session continuous time—all dimensions follow a 5-point Likert scale measurement framework. The total physical activity score is calculated as: frequency × intensity × (duration − 1), with scores ranging from 0 to 100. Scores of ≤19 indicate low physical activity, 20–42 indicate moderate activity, and ≥43 indicate high activity, with higher scores reflecting greater engagement in physical activity. The scale has been validated via psychometric indicators to possess good cross-cultural applicability and measurement stability.

Reliability was assessed by measuring internal consistency using both Cronbach’s *α* and McDonald’s *ω*. A Cronbach’s *α* value ranging from 0.5 to 0.7 indicates an acceptable level of reliability (Jiang et al., 2010), while McDonald’s *ω*—estimated via the omega function in the MBESS package for R (Dunn et al., 2014), with a value of 0.70 or above considered acceptable. In this study, the PARS-3 demonstrated a Cronbach’s *α* of 0.561 and a McDonald’s *ω* of 0.728. This relatively low Cronbach’s *α* may be because Cronbach’s *α* is sensitive to the number of items (Streiner et al., 2015). Critically, The McDonald’s *ω* coefficient is 0.728, indicating good reliability and confirming the scale’s sound consistency. Additionally, the K-S nonparametric test confirmed that the data distribution was normal (*p* < 0.001, df = 1,064).

#### 2.2.2 Adolescent Resilience Scale

The Adolescent Resilience Scale, developed by Hu and Gan (2008), consists of 27 items that measure five subdimensions: goal focus, emotional control, positive cognition, family support, and interpersonal assistance. Twelve items are reverse-scored, and all items use a 5-point Likert scale, with higher scores reflecting higher levels of psychological resilience. In this study, the scale demonstrated excellent internal consistency (Cronbach’s *α* = 0.932; McDonald’s *ω* = 0.941), with subdimension Cronbach’s *α* coefficients ranging from 0.745 (positive cognition) to 0.839 (emotional control). Confirmatory factor analysis confirmed good structural validity (*χ*^2^/df = 4.331, CFI = 0.914, TLI = 0.897, IFI = 0.915, RMSEA = 0.056, SRMR = 0.021).

### 2.3 Deep neural network model

Deep neural networks (DNN) (Shrestha and Mahmood, 2019) are deep learning models composed of multiple layers of neurons, capable of identifying complex correlations between variables through hierarchical feature extraction and nonlinear transformations. This makes DNNs especially suited for analyzing nonlinear relationships and multi-variable interactions that traditional linear models cannot capture, providing technical support for exploring the complex mechanisms linking physical exercise and psychological resilience. PyTorch (Jha and Pillai, 2021), an open-source deep learning framework, offers flexible dynamic computation graphs and a comprehensive library of neural network modules, enabling the construction of custom architectures, model training, and parameter optimization. It is widely used for regression and classification tasks in deep learning.

For this study, a DNN model was built using PyTorch, consisting of three fully connected layers: the input layer feeds data into the first fully connected layer with 128 neurons, using the ReLU activation function and 0.3 Dropout regularization to reduce overfitting. Data is then passed to a second fully connected layer with 64 neurons, incorporating ReLU activation and 0.3 Dropout. The third fully connected layer contains 32 neurons with ReLU activation but no Dropout. Finally, data are passed to an output layer with a single neuron to generate the final prediction. The ReLU activation function improves the network’s ability to model nonlinear relationships, while Dropout regularization helps prevent overfitting. For feature engineering, second-order polynomial feature expansion (PolynomialFeatures, degree = 2) was applied to capture complex interactions between variables, and StandardScaler was used to standardize the data, ensuring consistent input scaling. Although DNNs inherently possess the ability to model nonlinear relationships, for the specific scenario of this study, second-order polynomial feature expansion provides two key supplements: firstly, it captures potential low-order nonlinear patterns in the data in advance, avoiding the increased risk of overfitting that would arise if DNNs had to learn such basic patterns through iterative “trial-and-error” via deep network layers; secondly, embedding such interpretable nonlinear features directly into the input layer can assist subsequent SHAP interpretability analysis in accurately identifying key influencing factors. This provides clearer feature clues for analyzing “how physical exercise affects psychological resilience,” rather than relying on the black-box feature extraction of DNNs. At last, model training was carried out using a 5-fold cross-validation framework (*n*_splits = 5), with the Adam optimizer (learning rate = 0.001) and L1Loss (Mean Absolute Error) as the loss function. Training lasted for a maximum of 500 epochs, with early stopping (patience = 50) employed to monitor and optimize the training process.

### 2.4 SHAP explainable machine learning model

The SHAP explainable model was used to quantify the contribution of each variable to prediction outcomes through feature contribution values. SHAP is a unified framework based on Shapley values from game theory, with the core function of assigning importance weights to each feature for individual predictions, thereby explaining model outputs. Its core formula is shown in Equation 2. The additive feature attribution model defines SHAP as a linear function of binary variables, approximating the original model output as the sum of individual feature contributions. In this formula, *z*′∈0,1*^M^* (where *M* represents the number of features) indicates the presence or absence of features, *φ*ᵢ denotes the contribution of the *i*-th feature, and *φ*₀ represents the baseline value. This framework unifies six existing interpretability methods, including LIME and DeepLIFT, clarifying their classification as additive feature attribution approaches.


(2) $$g(z\prime )=\phi_{0}+\sum_{i=1}^{M}\phi_{i}z_{i}^{\prime }$$

The calculation of SHAP values strictly follows three fundamental axioms: local accuracy, missingness, and consistency (Molnar, 2022), and represents the unique solution that satisfies these axioms. Its calculation formula, shown in Equation 3, achieves quantification by averaging the contribution differences across all feature subsets with weighted averaging.


(3) $$\phi_{i}(f,x)=\sum_{z\prime \subseteq x\prime }\frac{|z\prime |!(M-|z\prime |-1)!}{M!}[f_{x}(z,\prime )-f_{x}(z\prime \backslash i)]$$

To ensure the explanation model is consistent with the original model output at the simplified input corresponding to the original input, as shown in Equation 4,


(4) $$f(x)=g(x\prime )=\phi_{0}+\sum_{i=1}^{M}\phi_{i}x\prime_{i}$$

where *x*′ = *hₓ*(*x*) denotes the simplified mapping of the original input *x*. This constraint ensures the fundamental accuracy of the explanation.

To efficiently approximate SHAP values, Kernel SHAP uses a specific weighted kernel function to perform weighted linear regression. The kernel function is presented in Equation 5,


(5) $$w_{x\prime }(z,\prime )=\frac{\left(M-1\right)}{\left(\frac{M}{|z\prime |})\;|z\prime |\;(M-|z\prime |\right)}$$

where (∣*z*′∣*^M^*) represents the combination number. This kernel function assigns appropriate weights to different feature subsets, ensuring that regression results align with the desired properties of SHAP values while improving computational efficiency.

### 2.5 Statistical methods

Statistical analysis was performed using SPSS 27.0 (IBM SPSS Inc.). Harman’s single-factor test was conducted to assess and control for common method bias in the questionnaire data. Descriptive analysis was performed for categorical variables, presenting counts (*n*) and proportions (%) to characterize the sample composition, with detailed results shown in Table 1. Spearman’s correlation analysis was used to examine the strength and direction of associations between variables. According to Cohen’s criteria for correlation strength, an *r* = 0 indicates no correlation, *r* = 0.20 to 0.50 indicates a weak to moderate correlation, and *r* = 0.50 to 0.80 indicates a strong correlation. Multiple linear regression analysis using ordinary least squares (OLS) was conducted to construct models examining the effects of each variable on psychological resilience and its subdimensions.

**Table 1:** Descriptive analysis results (*N* = 1,064).

Variable name	Variable category	Number (*n*)	Proportion (%)
Grade	1 = 7th grade	308	28.9
	2 = 8th grade	366	34.4
	3 = 9th grade	388	36.5
Age	1 = 11 years old	3	0.3
	2 = 12 years old	10	0.9
	3 = 13 years old	219	20.6
	4 = 14 years old	498	46.8
	5 = 15 years old	334	31.4
Gender	0 = female	496	53.3
	1 = male	567	46.6
Only child	0 = no	621	58.4
	1 = yes	442	41.5
Class cadre	0 = no	621	58.4
	1 = yes	442	41.5
Origin	0 = rural	473	44.5
	1 = urban	588	55.3

The PyTorch open-source deep learning framework was used to construct a DNN model based on the collected sample data, enabling the capture of nonlinear relationships between variables. To enhance model interpretability, SHAP values were used to quantify the contribution of each feature to model outputs, thereby clarifying the model’s predictive logic and variable interaction mechanisms. A significance level of *α* = 0.05 was set, with *p* < 0.05 indicating statistical significance.

## 3 Results and analysis

### 3.1 Common method bias

Data collected through questionnaires may be prone to common method bias due to factors such as measurement context, questionnaire instructions, and response contextual effects (Zhou and Long, 2004). To address this, Harman’s single-factor test was performed as a post-hoc assessment. All items from the PARS-3 and the Adolescent Resilience Scale were combined, and an unrotated exploratory principal component analysis was conducted. The results revealed the extraction of six factors with eigenvalues greater than 1, with the first factor accounting for 33.805% of the total variance, which is below the 40% threshold typically associated with significant common method bias. Therefore, no substantial common method bias was found in this study.

### 3.2 Descriptive analysis

As shown in Table 1, the sample consisted of 1,064 junior high school students, with the following demographic characteristics: In terms of grade distribution, ninth-grade students represented the largest group, followed by eighth-grade students. The age distribution was predominantly in the 14–15 years range, with students aged 11–12 accounting for less than 1%. Gender distribution showed a slight skew toward males. A larger proportion of participants were non-only children compared to only children. The number of non-class cadres was slightly higher than that of class cadres. Additionally, urban students made up a slightly larger proportion than rural students.

### 3.3 Correlation analysis

Table 2 presents the results of the correlation analysis. Psychological resilience and all its subdimensions showed significant positive correlations with physical exercise-related indicators, including intensity, duration, and frequency. In contrast, psychological resilience and its subdimensions displayed significant negative correlations with demographic characteristics such as grade and age.

**Table 2:** Correlation analysis results.

Variable	Grade	Age	Gender	Only child	Class cadre	Origin	Intensity	Duration	Frequency	Goal focus	Emotional control	Positive cognition	Family support
Age	0.502**												
Gender	−0.013	−0.037											
Only child	−0.021	−0.077*	−0.015										
Class cadre	0.02	0.011	−0.032	−0.006									
Origin	0.220**	0.025	−0.02	0.244**	0.001								
Intensity	−0.021	−0.073*	0.234**	0.04	0.086**	0.027							
Duration	−0.051	−0.067*	0.178**	−0.042	0.085**	0.020	0.437**						
Frequency	0.170**	0.088**	0.06	−0.029	0.035	0.191**	0.046	0.144**					
Goal focus	−0.119**	−0.129**	0.078*	0.088**	0.100**	0.110**	0.207**	0.212**	0.104**				
Emotional control	−0.130**	−0.098**	0.098**	0.057	0.027	0.077*	0.191**	0.216**	0.014	0.625**			
Positive cognition	−0.150**	−0.140**	0.056	0.006	0.122**	−0.034	0.233**	0.278**	0.119**	0.625**	0.488**		
Family support	−0.215**	−0.188**	0.012	0.079**	−0.008	0.018	0.117**	0.141**	−0.024	0.606**	0.611**	0.481**	
Interpersonal assistance	−0.103**	−0.134**	−0.004	0.103**	0.048	0.086**	0.153**	0.156**	0.014	0.560**	0.647**	0.470**	0.607**
Psychological resilience	−0.171**	−0.163**	0.049	0.079**	0.064*	0.071*	0.207**	0.236**	0.037	0.818**	0.852**	0.697**	0.820**

*indicates that the correlation coefficient or statistic is significant at the *p* < 0.05 level, **indicates significance at the *p* < 0.01 level, and ***indicates significance at the *p* < 0.001 level.

### 3.4 Multiple linear regression analysis

**Figure 1:**
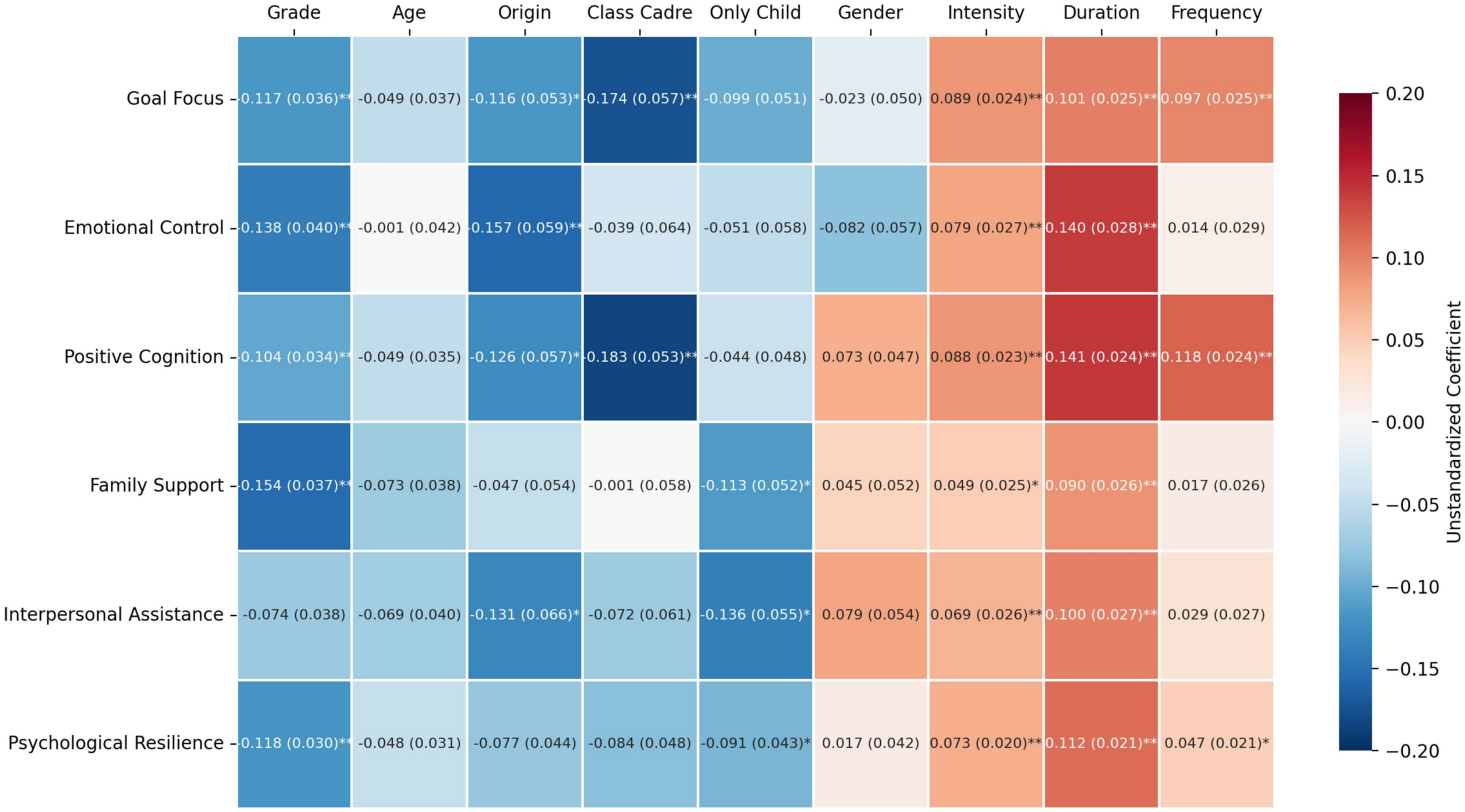
Correlation matrix of psychological resilience dimensions with demographic and exercise variables. This heatmap presents the unstandardized coefficients (B) along with their standard errors in parentheses, showing the correlations between various demographic and exercise-related variables (e.g., Grade, Age, Gender, Exercise Intensity, Duration, Frequency) and the subdimensions of psychological resilience (goal focus, emotional control, positive cognition, family support, interpersonal assistance, psychological resilience). Statistical significance is indicated by asterisks (*p < 0.05, **p < 0.01, ***p < 0.001).

A multicollinearity test showed that the variance inflation factor (VIF) for all independent and control variables was below 3, indicating no issues with multicollinearity. The model passed the overall significance test. Multiple linear regression (see Figure 1) results revealed that psychological resilience, as well as the subdimensions of interpersonal assistance, positive cognition, emotional control, and goal focus, were associated with demographic variables (such as grade and only-child status) and exercise variables (including intensity, duration, and frequency) to varying degrees. Notably, certain demographic variables had significant negative associations with psychological variables; for example, family support showed a negative correlation with psychological resilience (*β* = −0.073, *p* < 0.05). In contrast, physical exercise-related variables generally exhibited significant positive correlations with psychological variables: exercise intensity was positively correlated with psychological resilience (*β* = 0.118, *p* < 0.001), exercise duration was positively correlated with positive cognition (*β* = 0.141, *p* < 0.001), and exercise frequency was positively correlated with emotional control (*β* = 0.140, *p* < 0.001). The significance and magnitude of the effects varied across different psychological subdimensions.

### 3.5 Deep neural network and SHAP analysis

After constructing the DNN model, an interpretability analysis was conducted for psychological resilience and its subdimensions. The integration of DNN with SHAP tools revealed a consistent ranking pattern in feature importance across all dimensions: “Grade^2^” and “Frequency^2^” consistently ranked in the top two positions, emerging as key features explaining the multidimensional impact of physical exercise on psychological resilience. The key features identified through the combination of DNNs and SHAP are exactly the quadratic features extracted via polynomial feature expansion. This indicates that this operation has effectively captured the key nonlinear patterns of how physical exercise influences psychological resilience, rather than generating redundant features. At the same time, it also proves that embedding such low-order nonlinear features in advance helps the model focus more efficiently on core influencing relationships, preventing the dilution of key information caused by DNNs over-learning irrelevant complex patterns. To better understand their operational mechanisms, this study conducted an in-depth exploration of the dependence plots for these two quadratic features. When generating a dependence plot for a primary feature, SHAP automatically identifies the feature with the strongest interactive relationship with the primary feature in the dataset and uses color coding to represent the magnitude of the interactive feature’s values, thereby enhancing the visualization of interaction effects.

#### 3.5.1 Overall psychological resilience dimension

**Figure 2:**
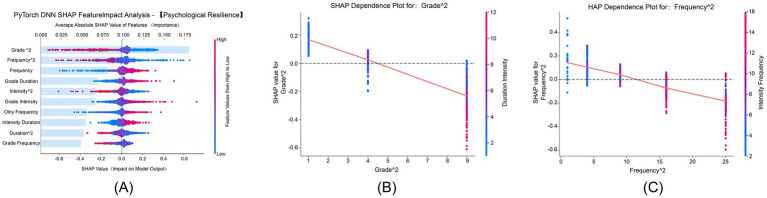
SHAP feature impact analysis for psychological resilience. This figure illustrates the average absolute SHAP values, which reflect the impact of each feature on the prediction of psychological resilience. The features analyzed include exercise intensity, duration, frequency, and demographic variables such as grade and only-child status. **(A)** Average SHAP values for each feature, indicating their relative importance in predicting psychological resilience. **(B)** SHAP dependence plot for Grade^2^, showing the interaction between grade level and psychological resilience. **(C)** SHAP dependence plot for Frequency^2^, highlighting the effect of exercise frequency on resilience outcomes.

Figure 2A illustrates that “Grade^2^” and “Frequency^2^” were ranked as the top two features, with importance values of 0.14 and 0.12, respectively, emphasizing the nonlinear cumulative effects of individual developmental stages and exercise frequency. Figure 2B shows that the grade quadratic term increases with advancing grade levels. Figure 2C demonstrates that, derived from the squaring of exercise frequency, the frequency quadratic term influenced psychological resilience as follows: high-frequency exercise had a weak inhibitory effect on psychological resilience due to excessive fatigue (SHAP value ≈ −0.6); moderate-to-high frequency exercise, when combined with high intensity, maximized its positive effect on psychological resilience (SHAP value up to 0.4); and low-frequency, low-intensity exercise consistently had a positive impact on psychological resilience (SHAP value ≈ 0.3).

#### 3.5.2 Positive cognition dimension

**Figure 3:**
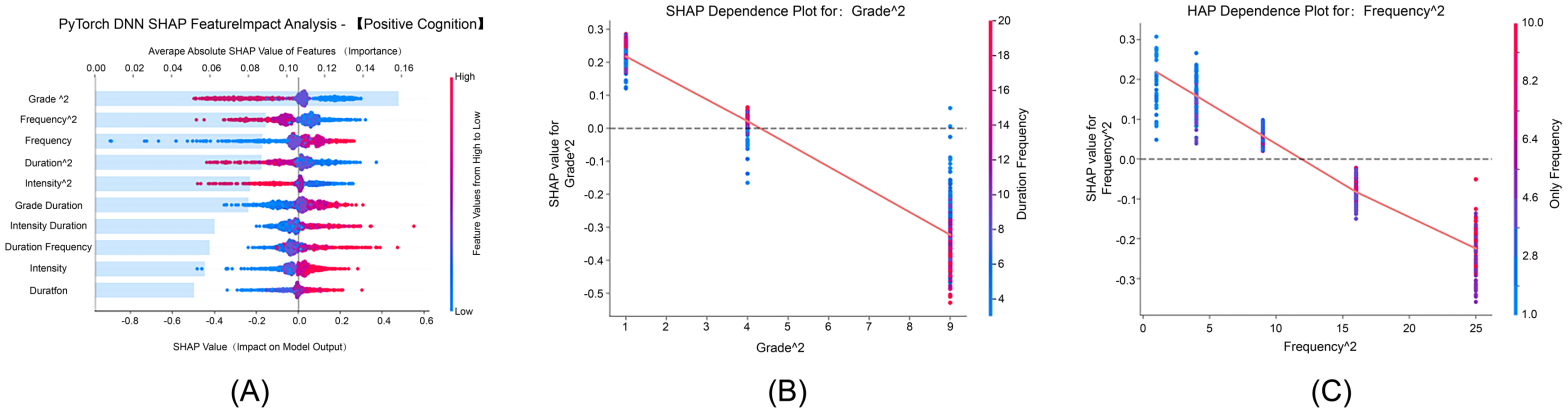
SHAP feature impact analysis for positive cognition. This figure focuses on how exercise characteristics and demographic variables influence the positive cognition dimension of psychological resilience. **(A)** SHAP analysis for positive cognition, ranking features by their contribution to the model. **(B)** SHAP dependence plot for Grade^2^, showing the relationship between grade level and positive cognition. **(C)** SHAP dependence plot for Frequency^2^, illustrating how exercise frequency influences positive cognition.

Figure 3A shows that “Grade^2^” and “Frequency^2^” had average absolute SHAP values of 0.175 and 0.150, respectively, ranking among the top features in terms of importance. SHAP values ranged from −0.2 to 0.4, indicating the presence of nonlinear interactions between physical exercise and positive cognition. Figure 3B demonstrates that the grade quadratic term increased with advancing grade levels, and its positive effect on positive cognition was progressively strengthened. Notably, increased exercise duration and frequency further amplified this effect, especially in higher-grade students. Figure 3C illustrates that the positive impact of the frequency quadratic term on positive cognition grew stronger with increasing exercise frequency, and the only-child status further enhanced the beneficial effects of high-frequency exercise.

#### 3.5.3 Interpersonal assistance dimension

**Figure 4:**
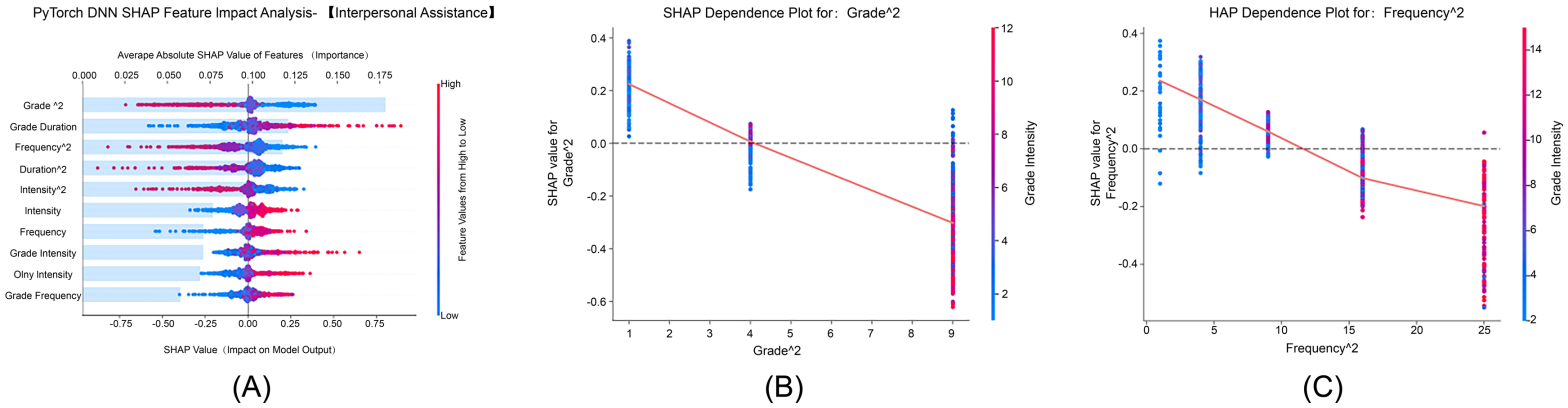
SHAP feature impact analysis for interpersonal assistance. This analysis shows how both demographic and exercise features contribute to the interpersonal assistance dimension of psychological resilience. **(A)** SHAP feature impact analysis for interpersonal assistance, showing the relative importance of different features. **(B)** SHAP dependence plot for Grade^2^, illustrating the effect of grade level on interpersonal assistance. **(C)** SHAP dependence plot for Frequency^2^, emphasizing the role of exercise frequency in improving interpersonal assistance.

Figure 4A shows that “Grade^2^” and “Frequency^2^” both had average absolute SHAP values of 0.175 and 0.150, respectively, serving as core features influencing interpersonal assistance. SHAP values ranged from −0.4 to 0.6, confirming the nonlinear interactions between physical exercise and interpersonal assistance. Figure 4B demonstrates that the grade quadratic term’s positive effect on interpersonal assistance strengthened with advancing grade levels, and this effect was further amplified by higher exercise intensity, particularly in higher-grade students. Figure 4C illustrates that the frequency quadratic term’s positive influence on interpersonal assistance grew stronger with increasing exercise frequency. The combination of higher grade levels and higher exercise frequency synergistically enhanced this effect, which was most pronounced in the high-frequency exercise group.

#### 3.5.4 Goal focus dimension

**Figure 5:**
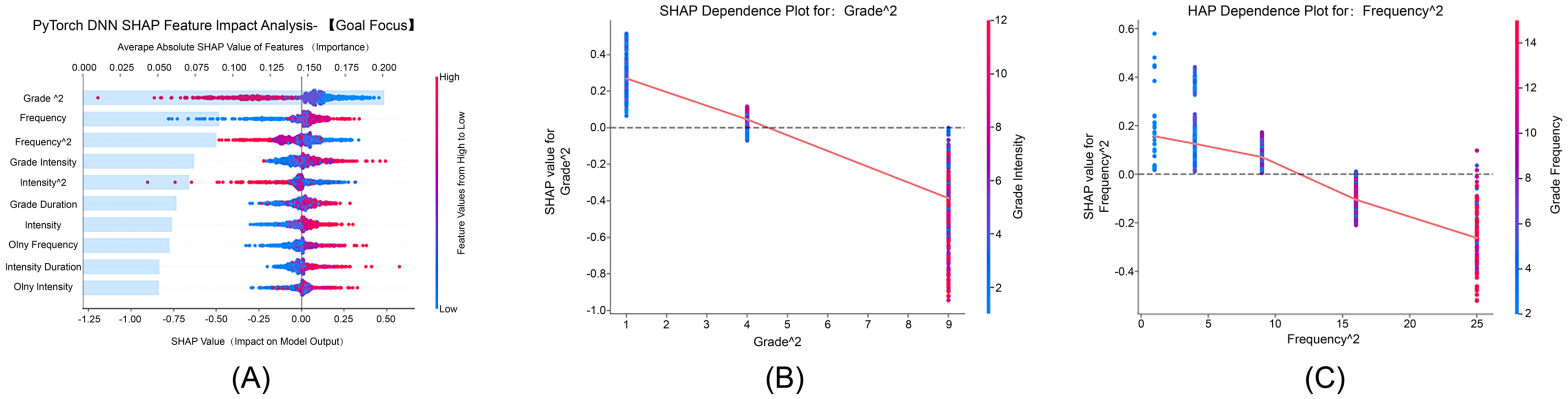
SHAP feature impact analysis for goal focus. This figure identifies the key factors that affect the goal focus dimension of psychological resilience. **(A)** SHAP feature impact for goal focus, with Grade^2^ and Frequency^2^ as the most significant features. **(B)** SHAP dependence plot for Grade^2^, showing how grade level influences goal focus. **(C)** SHAP dependence plot for Frequency^2^, illustrating the positive impact of higher exercise frequency on goal focus.

Figure 5A shows that “Grade^2^” and “Frequency^2^” had average absolute SHAP values of 0.25 and 0.20, respectively, emerging as the primary features influencing goal focus. SHAP values ranged from −0.2 to 1.0, indicating significant nonlinear interactions between physical exercise and goal focus. Figure 5B demonstrates that the grade quadratic term’s positive effect on goal focus gradually increased with advancing grade levels, and this effect was further enhanced by higher exercise intensity, reaching its peak in higher-grade students. Figure 5C illustrates that the positive impact of the frequency quadratic term on goal focus grew stronger with increasing exercise frequency. The interaction between higher grade levels and higher exercise frequency further amplified this effect, which was most pronounced in the high-frequency exercise group.

#### 3.5.5 Family support dimension

**Figure 6:**
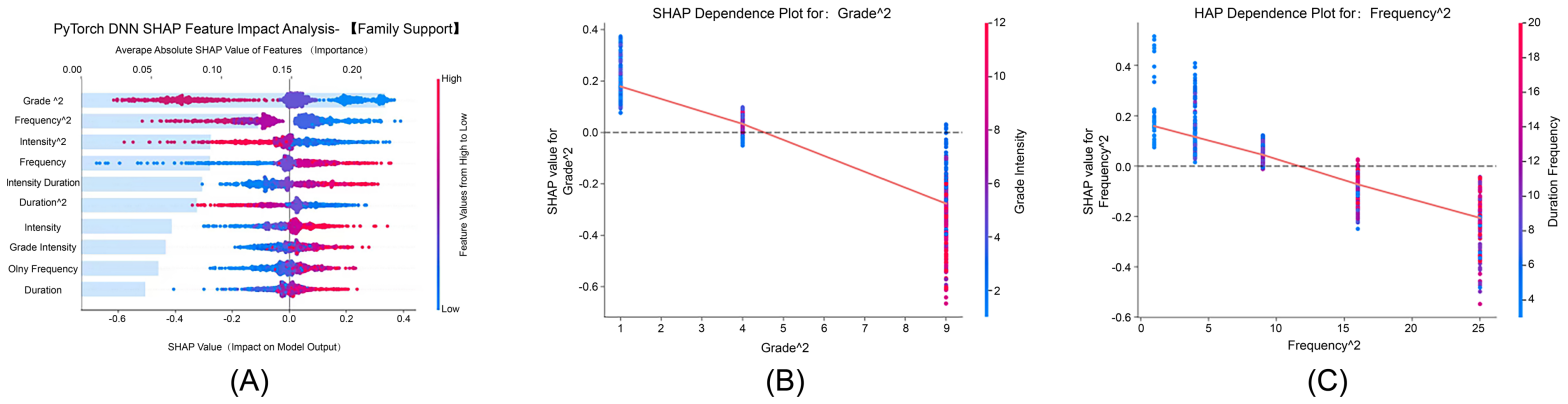
SHAP feature impact analysis for family support. This SHAP analysis explores how grade level and exercise characteristics influence the family support dimension of psychological resilience. **(A)** SHAP feature impact analysis for family support, showing the contribution of each feature. **(B)** SHAP dependence plot for Grade^2^, showing the effect of grade level on family support. **(C)** SHAP dependence plot for Frequency^2^, illustrating how exercise frequency influences family support.

Figure 6A shows that “Grade^2^” and “Frequency^2^” had average absolute SHAP values of 0.175 and 0.150, respectively, ranking among the top features in terms of importance. SHAP values ranged from −0.2 to 0.6, indicating nonlinear interactions between physical exercise and family support. Figure 6B demonstrates that the grade quadratic term’s positive effect on family support increased with advancing grade levels, and this effect was further amplified by higher exercise intensity, particularly in higher-grade students. Figure 6C illustrates that the positive influence of the frequency quadratic term on family support strengthened with increasing exercise frequency. The combination of longer exercise duration and higher frequency synergistically enhanced this effect, which was most pronounced in the high-frequency exercise group.

#### 3.5.6 Emotional control dimension

**Figure 7:**
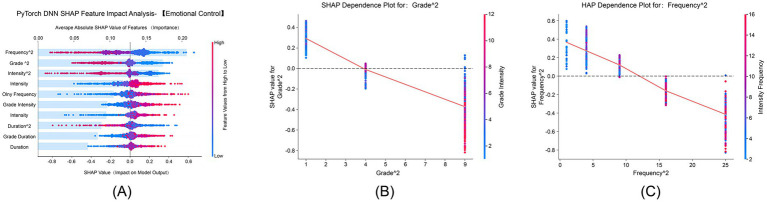
SHAP feature impact analysis for emotional control. This analysis shows the contribution of both demographic and exercise-related features to emotional regulation in the emotional control dimension. **(A)** SHAP feature impact for emotional control, identifying the most influential features. **(B)** SHAP dependence plot for Grade^2^, showing how grade level interacts with emotional control. **(C)** SHAP dependence plot for Frequency^2^, illustrating how frequency of exercise impacts emotional control.

Figure 7A shows that “Grade^2^” and “Frequency^2^” had average absolute SHAP values of 0.25 and 0.20, respectively, serving as core features influencing emotional control. SHAP values ranged from −1.0 to 1.0, confirming significant nonlinear interactions between physical exercise and emotional control. Figure 7B demonstrates that the grade quadratic term’s positive effect on emotional control gradually increased with advancing grade levels, and this effect was further amplified by higher exercise intensity, peaking in higher-grade students. Figure 7C illustrates that the positive impact of the frequency quadratic term on emotional control strengthened with increasing exercise frequency. The interaction between higher exercise intensity and higher frequency further enhanced this effect, which was most pronounced in the high-frequency exercise group.

## 4 Discussion

This study combined multiple linear regression, deep neural networks, and the SHAP explainable model to systematically explore the associations between physical exercise characteristics, demographic variables, and adolescent psychological resilience (including its five subdimensions). The core findings are as follows: the intensity, duration, and frequency of physical exercise were significantly positively correlated with adolescent psychological resilience and its subdimensions; grade level and only-child status exhibited significant interactive effects with physical exercise, while the gender × exercise interaction effect was not statistically significant; the SHAP model realized the quantification of feature contributions and visualization of interaction mechanisms, effectively addressing the interpretability limitations of traditional machine learning.

### 4.1 Main effects of physical exercise on the subdimensions of junior high school students’ psychological resilience

The main effects of physical exercise on the subdimensions of psychological resilience are specifically manifested as follows: exercise intensity is a prominent positive correlate of psychological resilience—moderate-to-high frequency exercise may exert its maximum benefits when paired with high intensity, whereas high-frequency exercise without sufficient intensity may lead to a weak inhibitory effect (presumably related to physical overfatigue), which is consistent with the research conclusions of Dunston et al. (2022). From the perspective of potential mechanisms, high-intensity exercise may activate the sympathetic nervous system (Szczegielniak and Krivošová, 2025), stimulating the release of endorphins and serotonin (Abdulaziz Muhsen and Abdulaziz Muhsen, 2020), which may in turn alleviate negative emotions induced by academic stress; meanwhile, through the cycle of “exposure to challenges and adaptive responses,” it may enhance individuals’ self-efficacy in coping with adversity (Donnelly et al., 2016).

For the positive cognition subdimension, exercise duration and frequency are core positive correlates, and only children may experience greater benefits from high-frequency exercise (Xia et al., 2024). Regular exercise can strengthen self-cognition through goal-setting experiences, and since only children lack feedback from siblings, they may rely more on such structured exercise feedback to build positive cognition. Physiologically, long-term exercise regulates prefrontal cortex function and optimizes information processing, laying a neural foundation for positive cognition (Krafft et al., 2014).

The improvement of the interpersonal assistance subdimension may be related to the social nature of collective exercise (Song X. L. et al., 2014). Teamwork and mutual support in sports (Pinto et al., 2021) may enhance communication skills, making frequent participants more likely to achieve higher scores. Older students tend to gain more benefits due to their increased need for peer approval, as social connections fostered through exercise may be internalized into supportive resources.

The enhancement of the goal focus subdimension may be associated with the synergistic effect of exercise intensity and frequency, and this effect may become more pronounced with age (Baumeister and Vohs, 2016). Regular moderate-to-high intensity exercise may strengthen self-regulation abilities, forming a cycle of “goal achievement—enhanced self-efficacy—strengthened goal focus.” This process may help older students alleviate the tendency of goal disengagement under academic pressure. Physiologically, moderate exercise optimizes the intrinsic organization of cognitive control networks (Krafft et al., 2014), supporting goal maintenance via neural mechanisms.

Family support may amplify the psychological benefits of physical exercise. Parents’ sports companionship, encouragement, and equipment support may reduce adolescents’ barriers to exercise, making it easier for them to gain a sense of accomplishment from exercise (Zhang et al., 2022). This synergistic effect may be more significant in lower-grade students, as they rely more on family support. From the perspective of Bourdieu’s cultural capital theory, family support—as emotion-based cultural capital—may be activated through parent-accompanied moderate-duration exercise, further strengthening exercise’s promoting effect on resilience subdimensions (e.g., family support, positive cognition) (Song G. W. et al., 2014).

The improvement of the emotional control subdimension may involve dual potential physiological and psychological mechanisms. Physiologically, regular exercise may promote the release of β-endorphins (Mahindru et al., 2023) to reduce anxiety and regulate the function of the hypothalamic–pituitary–adrenal (HPA) axis—moderate-to-high intensity exercise may reduce blunted cortisol responses, while a regular frequency of 3–5 sessions per week may maintain this physiological regulatory effect, thereby potentially enhancing emotional control and goal focus (Nishimi et al., 2022). Psychologically, high-intensity exercise may release negative emotions, and the emotional regulation skills acquired through exercise may be transferable, helping to improve long-term emotional control efficiency.

### 4.2 Interactive effects between demographic variables and physical exercise

Regarding the interactive effects between demographic variables and physical exercise, grade serves as a core moderator of the psychological benefits of exercise during the junior high school stage. Seventh-grade students, with relatively low academic pressure and abundant psychological resources, may improve resilience through low-frequency, low-intensity regular exercise (Xing et al., 2023), a process that may be driven by the physical pleasure and sense of achievement brought by exercise. Eighth-grade students, entering early adolescence, experience rapid development of self-awareness, fluctuating peer relationships, and reduced emotional stability, which may weaken or offset the positive effects of exercise. Ninth-grade students face intense examination pressure that may exert a weak inhibitory effect on resilience, but high-intensity exercise may counteract part of this negative impact through a stress compensation effect (Song G. W. et al., 2014).

The interaction between only-child status and exercise is mainly reflected in the positive cognition and goal focus subdimensions. Lacking sibling interactions during childhood (Gutgesell and Payne, 2004; He, 2020), only children may rely more on external feedback, so the regular goal-setting and feedback loops of high-frequency exercise may be more beneficial to their positive cognition. In contrast, non-only children may develop mature goal-regulation abilities through long-term sibling interactions (McHale et al., 2012) and only need moderate-to-low frequency exercise.

Notably, this study did not support the hypothesized gender × exercise interaction effect (inconsistent with H2), which may be explained by three factors: (1) homogeneous school PE contexts—current curricula lack gender-differentiated content, with boys and girls engaging in similar exercise types, intensities, and social scenarios (Han et al., 2025), eliminating potential gender differences in exercise-resilience mechanisms; (2) sample behavioral tendencies—while gender distribution was balanced, girls preferred low-intensity activities (e.g., walking, rope skipping) and boys favored high-intensity sports (e.g., basketball, soccer) (Li, 2023), offsetting inherent gender interaction effects; (3) immature gender role identity among junior high school students (Peyer et al., 2022)—weak sociocultural gender expectations on exercise experiences hinder significant gender-based interactions.

### 4.3 Explanatory value of the SHAP model and quantitative mechanisms of exercise effects

The SHAP model realized the quantification and visualization of the associations between physical exercise and psychological resilience, revealing nonlinear patterns that are difficult for traditional linear models to capture. The dominance of ‘Grade^2^’ and ‘Frequency^2^’ (top two features across all resilience dimensions) reflects their core role in explaining exercise-resilience associations. Specifically, the high contribution of ‘Grade^2^’ confirms a non-linear grade-resilience trajectory (‘weak promotion—fluctuation—weak inhibition’), aligning with the ‘qualitative leap’ theory of adolescent psychological development (Mastorci et al., 2024).

The core role of “Frequency^2^” is manifested as follows: low-frequency exercise has a weak positive effect, moderate-frequency exercise shows a significantly enhanced effect, and excessively high-frequency exercise may produce an inhibitory effect due to physical exhaustion. This suggests that exercise benefits have a critical threshold, beyond which they may diminish (Mastorci et al., 2024). SHAP dependence plots across dimensions clarified three key interaction logics: older students may need high-intensity exercise to counteract the negative impact of academic pressure; only children may rely on high-frequency exercise to obtain external goal feedback, while non-only children develop mature cognitive regulatory abilities through family interactions (McHale et al., 2012); moderate-to-high frequency exercise can only maximize its benefits when paired with sufficient duration, a conclusion consistent with existing literature (Parfitt and Hughes, 2009; Eston et al., 2012).

### 4.4 Study limitations

This study has certain limitations: the cross-sectional design can only reveal the correlational relationship between physical exercise and psychological resilience, but cannot clarify the direction of causality and the action path, nor can it rule out the reverse causality hypothesis. Future studies could be improved through longitudinal follow-ups of at least 12 months and small-scale randomized controlled trials.

There was a significant group bias at the sample level: the proportion of urban students and non-only children was excessively high, while rural students and only children were severely underrepresented. This bias is directly linked to the study’s core variables: rural and urban adolescents differ in exercise resources and family support models, while only children and non-only children have distinct exercise feedback needs. These differences limit the generalizability of findings to urban non-only children. In the future, stratified sampling should be adopted to balance the sample proportion according to dimensions such as urban–rural background and only-child status.

There were also deficiencies in variable control and analysis: key confounding variables such as exercise type, academic stress (Leng et al., 2025), and family economic status (Lou et al., 2024) were not measured in a refined manner. Among them, adolescent academic stress was only mentioned but not quantified, and stress of different intensities may moderate the effect of exercise; variables such as family economic status may obscure or exaggerate the real correlation. Meanwhile, the SHAP analysis only focused on bivariate interactions and failed to explore the complex three-way interaction among intensity, duration, and frequency, leading to an insufficiently in-depth interpretation of the mechanism. In the future, a multi-dimensional structural equation model should be constructed to incorporate relevant variables and interaction effects.

The findings of this study can only serve as a reference for understanding the association between physical exercise and the development of adolescent resilience, and flexible adjustments should be made in combination with specific scenarios and group characteristics when applying these findings.

## 5 Conclusion

In summary, physical exercise intensity, duration, and frequency are significantly positively associated with junior high school students’ psychological resilience and its five subdimensions (goal focus, emotional control, positive cognition, family support, and interpersonal assistance). Demographic variables, including grade and only-child status, may interact with physical exercise characteristics to collectively shape these resilience dimensions, while the hypothesized gender × exercise interaction effect was not statistically significant. The SHAP model effectively quantified the contribution of each feature and visualized potential nonlinear interaction mechanisms, demonstrating its value in interpreting complex machine learning results. These findings offer empirical insights for designing differentiated physical exercise interventions: low-intensity regular exercise is recommended for seventh-grade students; high-intensity, moderate-frequency exercise may be beneficial for ninth-grade students; and only children may gain additional cognitive benefits from structured, high-frequency exercise programs.

It is important to note that due to the cross-sectional design of this study, the observed associations cannot be interpreted as causal relationships, and the direction of influence between physical exercise and psychological resilience remains to be clarified. Future longitudinal studies and randomized controlled trials are needed to further verify the potential causal pathways and long-term effects of physical exercise on adolescent psychological resilience. Nevertheless, the current findings provide preliminary empirical references for understanding the complex relationships between physical exercise and adolescent psychological resilience, and offer practical implications for developing targeted exercise strategies to support adolescent mental health.

## Data Availability

The raw data supporting the conclusions of this article will be made available by the authors, without undue reservation.
